# A comprehensive overview of clinical research on dexmedetomidine in the past 2 decades: A bibliometric analysis

**DOI:** 10.3389/fphar.2023.1043956

**Published:** 2023-02-14

**Authors:** Hao Kong, Mo Li, Chun-Mei Deng, Yu-Jia Wu, Shu-Ting He, Dong-Liang Mu

**Affiliations:** Department of Anesthesiology and Critical Care Medicine, Peking University First Hospital, Beijing, China

**Keywords:** dexmedetomidine, bibliometric study, VOSviewer, Citespace, sedation

## Abstract

**Introduction:** Dexmedetomidine is a potent, highly selective α-2 adrenoceptor agonist with sedative, analgesic, anxiolytic, and opioid-sparing properties. A large number of dexmedetomidine-related publications have sprung out in the last 2 decades. However, no bibliometric analysis for clinical research on dexmedetomidine has been published to analyze hot spots, trends, and frontiers in this field.

**Methods:** The clinical articles and reviews related to dexmedetomidine, published from 2002 to 2021 in the Web of Science Core Collection, were retrieved on 19 May 2022, using relevant search terms. VOSviewer and CiteSpace were used to conduct this bibliometric study.

**Results:** The results showed that a total of 2,299 publications were retrieved from 656 academic journals with 48,549 co-cited references by 2,335 institutions from 65 countries/regions. The United States had the most publications among all the countries (n = 870, 37.8%) and the Harvard University contributed the most among all institutions (n = 57, 2.48%). The most productive academic journal on dexmedetomidine was *Pediatric Anesthesia* and the first co-cited journal was *Anesthesiology*. Mika Scheinin is the most productive author and Pratik P Pandharipande is the most co-cited author. Co-cited reference analysis and keyword analysis illustrated hot spots in the dexmedetomidine field including pharmacokinetics and pharmacodynamics, intensive care unit sedation and outcome, pain management and nerve block, and premedication and use in children. The effect of dexmedetomidine sedation on the outcomes of critically ill patients, the analgesic effect of dexmedetomidine, and its organ protective property are the frontiers in future research.

**Conclusion:** This bibliometric analysis provided us with concise information about the development trend and provided an important reference for researchers to guide future research.

## 1 Introduction

Dexmedetomidine, a highly selective α-2 adrenoreceptor agonist, has broad-spectrum effects including sedative, analgesic, and anxiolytic properties with minimal respiratory depression (Carollo et al., 2008). It was approved as a short-term (<24 h) sedative agent in 1999, for sedation in non-tracheal intubation patients in 2008, and for sedation during general anesthesia, endotracheal intubation, and mechanical ventilation in 2009 by the U.S. Food and Drug Administration (FDA) (Paris and Tonner, 2005; Bao and Tang, 2020). Sedation with dexmedetomidine was associated with shorter durations of mechanical ventilation and intensive care unit (ICU) stay, lower risk of delirium, and better postoperative cognitive function (Mo and Zimmermann, 2013; Buckley et al., 2021; Liu et al., 2022). The analgesic property was also identified as supplementation to intravenous analgesic, peripheral nerve block, and intrathecal anesthesia (Schnabel et al., 2013; Mohamed et al., 2014; Shen et al., 2020). The anxiolytic effect promoted the use of dexmedetomidine in children as premedication (Peng et al., 2014). In recent years, its organ-protective effects, *via* reducing the inflammatory response and activating antiapoptotic signaling pathways, have caught the attention of researchers and clinicians (Bao and Tang, 2020). In addition, dexmedetomidine exerts its versatile applications in decreasing the occurrences of postoperative nausea and vomiting (Zhang et al., 2022), attenuating shivering (Sween et al., 2021), improving sleep quality (Wu et al., 2016), relieving sore throat (Liu et al., 2021), and preventing catheter-related discomfort (Kim et al., 2015).

Over the past 20 years, dexmedetomidine was extensively used in clinical practice and raised a hot research topic. Bibliometric analysis is often used to comprehensively summarize the contributions of scientific publications based on constructing the citation graph, a network representing the citations of different documents. In addition, it is also used for exploring the impact of researchers and a special paper within a specific research field. To the best of our knowledge, there is no bibliometric analysis for clinical research on dexmedetomidine. We conduct this analysis to provide a systematic overview of the evolutionary process, hot spots, and future directions of dexmedetomidine research. It will help researchers to further understand global research trends and provide enlightenment for future research in drug development and clinical application.

## 2 Materials and methods

### 2.1 Data source and search strategy

Bibliometric data about dexmedetomidine from 2002 to 2021 was obtained from the Science Citation Index Expanded (SCI-EXPANDED; 1900–2021) in the Web of Science Core Collection (WoSCC) database on May 19 2022. The search terms were “[Topic (dexmedetomidine) OR Topic (MPV-1440) OR Topic (MPV 1440) OR Topic (MPV1440) OR Topic (Precedex) OR Topic (Dexmedetomidine Hydrochloride) OR Topic (Hydrochloride, Dexmedetomidine)] Not [ (Topic (animal*) OR Topic (cell) OR Topic (*in-vitro*) OR Topic (rat*) OR Topic (dog*) OR Topic (mice) OR Topic (mouse) OR Topic (pig*) OR Topic (horse*) OR Topic (monkey*) OR Topic (veterinary)) Not (Topic (human) OR Topic (humans))]]”, the period of publication ranging from 2002 to 2021. The search was performed on a single day to avoid bias caused by daily database updates.

### 2.2 Inclusion and exclusion criteria

In the present study, clinical original articles and reviews written in English were included. Studies were excluded if they met any of the following items: 1) meeting abstracts, letters, comments, and editorials; 2) no abstract or the digital object identifier (DOI) number; 3) unavailable with full text; 4) translated versions of articles or reviews; 5) retracted publication; 6) duplicate literature.

### 2.3 Study selection and data management

Two groups of reviewers (M Li and C-M Deng and Y-J Wu and S-T He) independently performed study selection and data extraction after standard training. Differences of opinion were settled by referral to a third group of reviewers (D-L Mu and H Kong). Title and abstracts were first screened to select the articles. Full texts were retrieved when necessary. For keywords with different expressions, we have processed them, leaving only one standardized keyword.

### 2.4 Data analysis

Publication characteristics were tabulated, including titles, authors, journal sources, keywords, affiliations of authors, and the continents and countries/regions to which the authors belong. VOSviewer (Version 1.6.18) software, a literature analysis and knowledge visualization software tool developed by van Eck and Waltman (2010) was utilized to construct and visualize the relationships among the most highly productive countries, research institutions, and author keywords. Publication characteristics, including year, authors, co-cited authors, countries, institutes, journal sources, co-cited journals, keywords, and co-cited references, were also analyzed. Co-cited authors are defined as the authors who are cited together. Co-cited references are references that have been co-cited in a set of publications. The colors of nodes and lines represent different clusters or years. In the VOSviewer network maps, different nodes indicate components, such as countries/regions, institutions, and journals. The sizes of the nodes reflect the number of studies or co-occurrence frequencies. The links between nodes represent the co-occurrence relationships, meanwhile, their sizes indicate the co-occurrence frequencies of the two nodes. The VOSviewer settings were as follows: counting method (full counting), and ignoring documents with a large number of authors (maximum number of authors per document is 25). While, thresholds T) of items (countries/regions, institutions, journals, authors, and references) were adopted based on special situations. CiteSpace was also a powerful and complementary science mapping analysis software, proposed by Professor Chen (2004). CiteSpace (5.8. R3) explores the tendencies and dynamics of scientific studies in a given research field, and we used it to detect the references and keywords with strong citation burstness to identify emerging topics. The CiteSpace parameters were as follows: link retaining factor (LRF = 3), look back years (LBY = 5), e for top N (e = 1), time span (2002–2021), years per slice 1), links (strength: cosine, scope: within slices), selection criteria (g-index: k = 20 for analyzing the co-cited references; g-index: k = 10 for analyzing the keywords), and minimum duration (MD = 3).

## 3 Results

A total of 3,690 dexmedetomidine-associated publications were identified from 2002 to 2021. Among them, 1,289 were excluded for not articles or review articles, 57 for not English, 42 for proceeding papers, one for retracted publication, and two for books. The remaining 2,299 were included in the final analysis. 1867 (81.2%) records were research articles and 432 (18.8%) records were review.

### 3.1 Annual growth trend of publications and total global citation score (TGCS)

**FIGURE 1:**
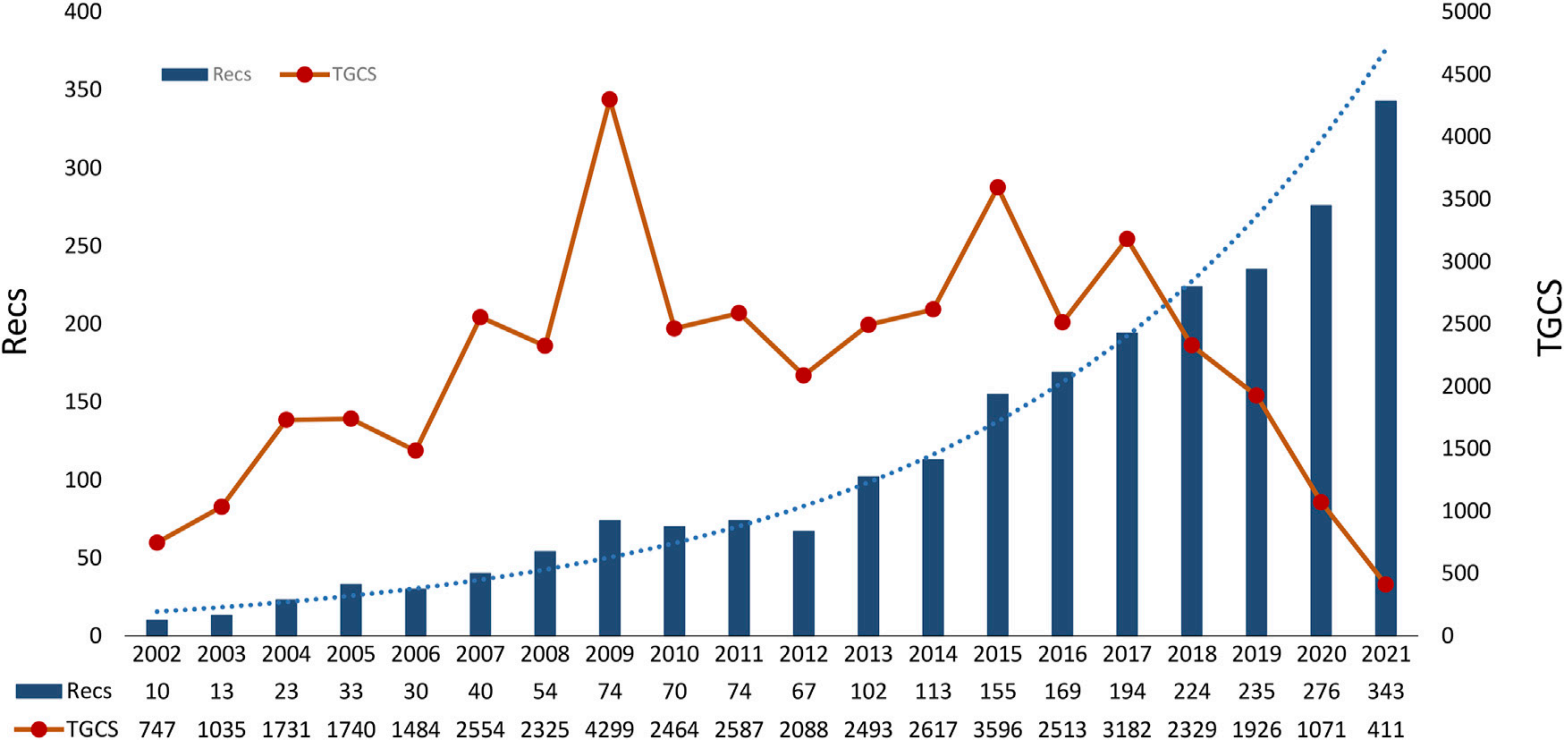
Timeline of publications and TGCS on dexmedetomidine. (TGCS refers to the total number of citations of all dexmedetomidine-related clinical publications in a certain year retrieved from the Web of Science Core Collection database). TGCS, total global citation score.

The annual output of dexmedetomidine-related studies increased steadily. From 2002 to 2012, the annual publications were less than 100. There was a significant increase from 2013 to 2018 and it reached the maximum in 2021 (n = 343) (Figure 1). The TGCS showed an upward trend during the period from 2002 to 2017, indicating the growing interest in dexmedetomidine-related research. It is worth noting that TGCS reached its peak in 2009 with the highest value of 4,299. From 2018, the TGCS decreased year by year.

### 3.2 Countries/regions and institutions analysis

**FIGURE 2:**
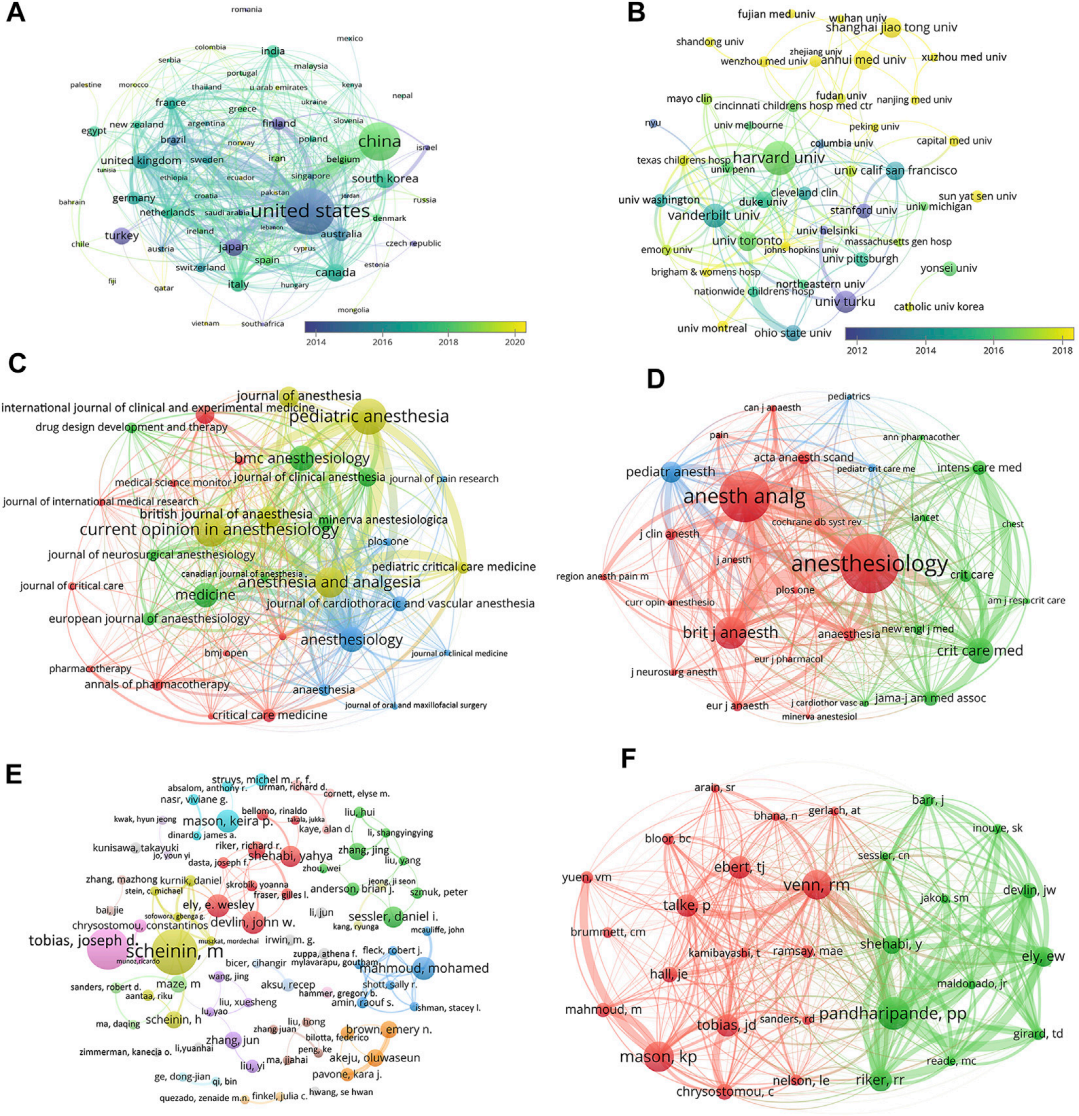
Academic collaboration between different countries/regions, institutions, journals, and authors in the dexmedetomidine research area. **(A)** Collaboration between different countries/regions (Threshold = 1) **(B)** Collaboration between different institutions (Threshold = 15) **(C)** Collaboration between different journals (Threshold = 13) (**D)** Collaboration between different co-cited journals (Threshold = 350) **(E)** Collaboration between different authors (Threshold = 5) **(F)** Collaboration between different co-cited authors (Threshold = 109).

Dexmedetomidine-related articles from 2002 to 2021 were mainly published by 65 countries/regions with 2,335 institutions. Table 1 showed the top 10 countries/regions and institutions involved in dexmedetomidine research. The top 10 countries/regions were distributed in four continents including four in Asia and three in Europe. The largest number of papers originated from the United States (870 publications), followed by China (575 publications). The countries like South Korea (119 publications), Canada (108 publications), and Japan (102 publications) have also made great contributions to the research of dexmedetomidine. Figure 2A, the network map of countries/regions, showed many active collaborations among them. For example, the United States has intense cooperation with China, Canada, Japan, the United Kingdom, and Finland. The color of nodes in Figure 2A also showed the number of publications in different countries over time. Articles from China are published later than those in the United States, Finland, Japan, and Turkey.

**TABLE 1:** The top 10 countries/regions and institutions involved in dexmedetomidine research from 2002 to 2021.

Rank	Country/Region	Count	Total link strength^a^	Centrality^b^	Rank	Institution	Count	Total link strength^a^	Centrality^b^
1	United States	870	299	0.44	1	Harvard University (United States)	57	143	0.07
2	China^c^	575	113	0.08	2	Vanderbilt University (United States)	40	155	0.12
3	South Korea	119	24	0.00	3	University of Turku (Finland)	37	63	0.03
4	Canada	108	104	0.10	4	Shanghai Jiao Tong University (China)	34	29	0.03
5	Japan	102	41	0.01	5	University of Toronto (Canada)	33	148	0.05
6	Turkey	97	5	0.00	6	Anhui Medical University (China)	32	11	0.02
7	United Kingdom^d^	83	143	0.16	7	University of California, San Francisco (United States)	31	79	0.04
8	Italy	65	102	0.07	8	Duke University (United States)	28	78	0.02
9	Australia	60	85	0.05	9	Ohio State University (United States of America)	27	43	0.02
10	Finland	55	42	0.03	10	Stanford University (United States)	25	44	0.01
						University of Pittsburgh (United States)		52	0.03
						Yonsei University (South Korea)		11	0.00

^a^ Made by VOSviewer, counting method was full counting, ignored documents co-authored by a large number of countries which the maximum number of countries per document was 25.

^b^ Made by CiteSpace, link retaining factor = 3.0, maximum links per node = 10, look back years = 5, e = 1.0, selection criteria was g-index which k = 25.

^c^ Including publications from the Chinese Mainland, Hong Kong, Macau, and Taiwan.

^d^ Including publications from England, Scotland, Northern Ireland, and Wales.

The top 10 institutions (including 12 centers, three tied for the 10th place) are distributed in five countries/regions, seven in the United States, two in China, one in Finland, one in Canada, and one in South Korea (Table 1). Harvard University, Vanderbilt University, and the University of Turku were the top three academic institutions with the highest publications (Table 1). Figure 2B showed close cooperation among American institutions. The Chinese institutions’ network is constructed independently in recent years.

### 3.3 Journals and Co-Cited academic journals

The 2,299 publications analyzed were published in 656 journals. The top 10 journals with the most publications account for 22.2% (511) of all articles (Table 2). Eight of the 10 journals are in the field of Anesthesiology. The top 30 journals (32/656, 3.07%) were used to construct the citation network map, Figure 2C. There are active citation relationships among *Anesthesiology*, *Anesthesia and Analgesia*, *Pediatric Anesthesia*, *British journal of anaesthesia*, and *Current Opinion in Anesthesiology*.

**TABLE 2:** The top 10 journals and co-cited journals of dexmedetomidine research from 2002 to 2021.

Rank	Journal	Counts	Rank	Co-cited journal	Citations
1	Pediatric Anesthesia	72	1	Anesthesiology	6,318
2	Current Opinion in Anesthesiology	68	2	Anesthesia and Analgesia	5,370
3	Anesthesia and Analgesia	60	3	British Journal of Anaesthesia	3,172
4	BMC Anesthesiology	51	4	Critical care medicine	2,459
5	Anesthesiology	50	5	Pediatric Anesthesia	1931
6	Medicine	49	6	JAMA	1,307
7	British Journal of Anaesthesia	45	7	Intensive Care Medicine	1,246
8	Journal of Anesthesia	41	8	Anaesthesia	1,216
9	Journal of Clinical Anesthesia	38	9	Acta Anaesthesiologica Scandinavica	1,160
10	International Journal of Clinical and Experimental Medicine	37	10	Critical Care	1,031

JAMA, journal of the american medical association.

JCR, journal citation reports.

Among 7,055 co-cited academic journals, 120 journals had co-citations over 100. The top 10 journals with the most co-citations were shown in Table 2. *Anesthesiology* had the most co-citations, followed by *Anesthesia and Analgesia*, and *British Journal of Anaesthesia*. Six journals are in the field of Anesthesiology, three are in the field of Critical Care Medicine. As one of the most influential medical journals, *JAMA* also ranks in the top 10 in the research field of dexmedetomidine. In the co-citation network constructed by the Top 30 journals, the top three most co-cited journals (*Anesthesiology*, *Anesthesia and Analgesia*, and *British Journal of Anaesthesia*) also have the most active co-cited relationships among them (Figure 2D).

### 3.4 Authors and Co-Cited authors

A total of 10,425 authors were involved in the dexmedetomidine-related studies. 78 authors published over five articles. Mika Scheininfrom the University of Turku, Finland published the most articles, followed by Joseph D Tobias from Nationwide Children’s Hospital, Columbus, Ohio, Missouri, United States (Table 3). From the co-authorship map (Figure 2E), close cooperation was observed among several authors, such as Scheinin M and Scheinin H; Shehabi Y, Riker RR, and Devlin JW.

**TABLE 3:** The top 10 authors of dexmedetomidine research from 2002 to 2021.

NO. Of articles			NO. Of Co-citation		
Rank	Authors	Count	Rank	Co-cited authors	Citations
1	Mika Scheinin (University of Turku, Finland)	24	1	Pratik P Pandharipande (Vanderbilt University School of Medicine, Nashville, Tennessee, United States)	415
2	Joseph D Tobias (Nationwide Children’s Hospital, Columbus, Ohio, Missouri, USA/The Ohio State University College of Medicine, Columbus, Ohio, United States)	22	2	Keira P Mason (Children’s Hospital Boston and Harvard Medical School, Boston, MA, United States)	379
3–6	John W Devlin (Northeastern University, Boston, MA, USA/Tufts Medical Center, Boston, MA, United States)	12	3	Richard M Venn (St George’s Hospital, London, United Kingdom)	375
	E Wesley Ely (Center for Health Services Research, Nashville, Tennessee, United States)		4	Richard R Riker (University of Vermont College of Medicine, United States)	287
	Mohamed Mahmoud (Cincinnati Children’s Hospital Medical Center, Cincinnati, Ohio, United States)		5	Joseph D Tobias (Nationwide Children’s Hospital, Columbus, Ohio, Missouri, USA/The Ohio State University College of Medicine, Columbus, Ohio, United States)	286
	Keira P Mason (Children’s Hospital Boston and Harvard Medical School, Boston, MA, United States)		6–7	Pekka Talke (University of California, San Francisco, CA, United States)	284
				Tomas J Ebert (The Medical College of Wisconsin and the VA Medical Center, Milwaukee, United States)	
7–8	Daniel I Sessler (The Cleveland Clinic-P77, Cleveland, Ohio, United States)	11	8	E Wesley Ely (Center for Health Services Research, Nashville, Tennessee, United States)	255
	Yahya Shehabi (Monash University, Melbourne, VIC, Australia)		9	Yahya Shehabi (Monash University, Melbourne, VIC, Australia)	233
9–10	Oluwaseun Akeju (Massachusetts General Hospital, Boston, MA, USA/Harvard Medical School, Boston, MA, United States)	9	10	Constantinos Chrysostomou (Children’s Hospital of Pittsburgh, Pittsburgh, PA, United States)	203
	Emery N. Brown (Massachusetts General Hospital, Boston, MA, United States)				
	Richard R Riker (University of Vermont College of Medicine, United States)				
	Harry Scheinin (University of Turku, Finland)				
	Mervyn Maze (University of California, San Francisco, California, United States)				

Co-cited authors are authors who have been co-cited in a range of publications. Among 35,043 co-cited authors, three authors had co-citations over 300. Pratik P Pandharipande had the most co-citations (n = 415) and ranked first, followed by Keira P Mason (n = 379) and Richard M Venn (n = 375) (Table 3). The top 30 authors were used to construct the co-citation picture. According to Figure 2F, Pandharipande PP has active co-cited relationships with Riker RR, Ely EW, Devlin JW, and Shehabi Y; Mason KP has strong co-cited relationships with Yuen VM and Mahmoud M; Venn RM has a close co-cited relationship with Talke P.

### 3.5 Co-cited reference analysis and burst analysis

**FIGURE 3:**
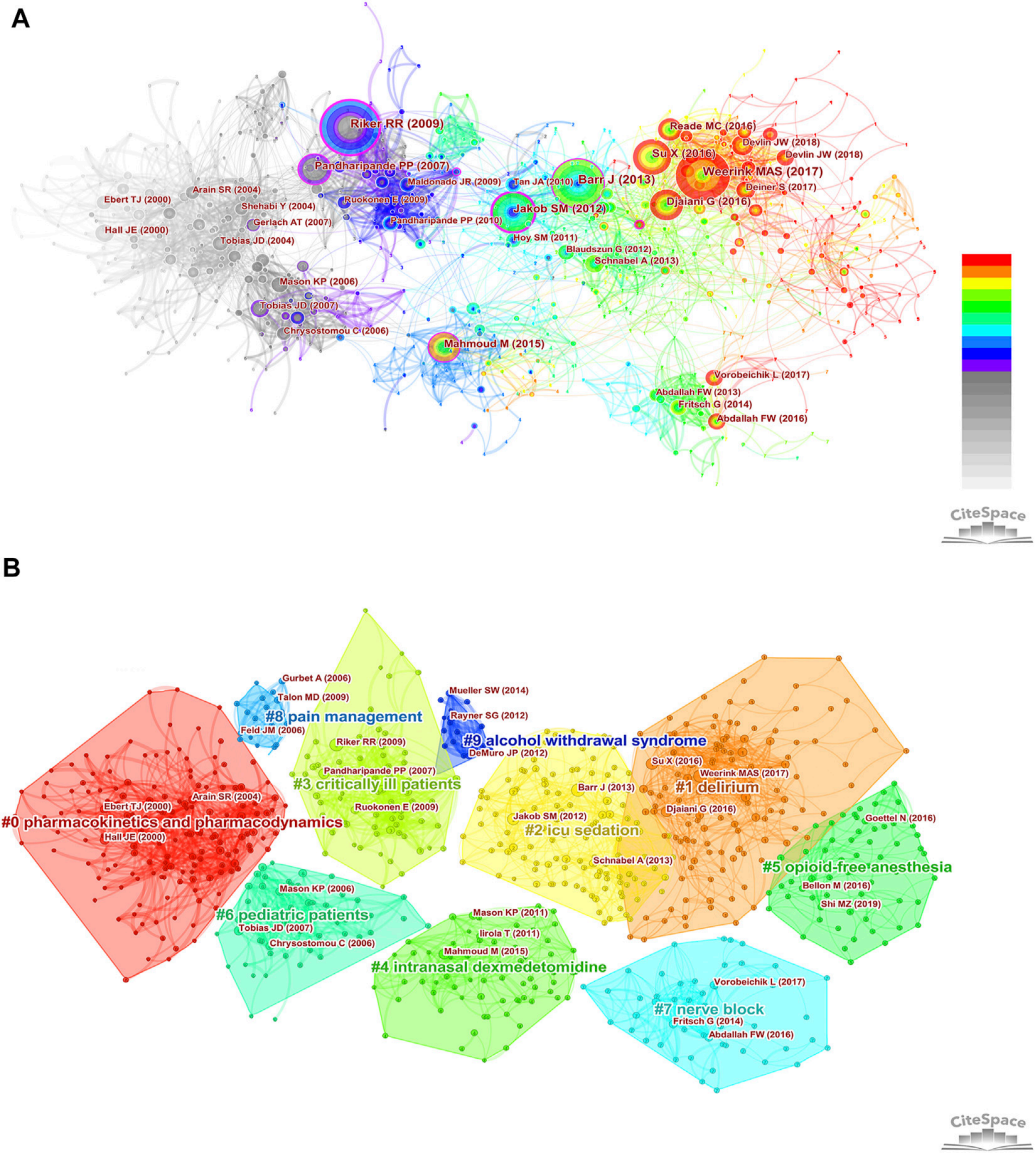
Co-cited references analysis. **(A)** The overall display diagram of the co-cited references. (The size of the nodes represents the co-cited times of one reference. Time is reflected by different colors: light gray refers to 2002 and red refers to 2021. The nodes with purple around refer to references with high centrality) **(B)** Cluster diagram of co-cited references (All the references belonging to one cluster are covered by regions with different colors. The top three most co-cited references were marked in each cluster.)

Co-cited references are those references, which are cited together by other publications. Among 2,299 dexmedetomidine-related publications, there were 48,549 cited references. Figure 3A showed the overall display diagram of the co-cited references. The nodes with purple around refer to references with high centrality. The articles by Riker et al. (2009), Jakob et al. (2012), and Barr et al. (2013) had the top three highest centralities (0.22, 0.22, and 0.17, respectively). The size of the nodes represents the co-cited times of one reference. We have presented the top 10 co-cited clinical references in Table 4. Six of 10 were randomized clinical trials, of which all focused on the effect of dexmedetomidine sedation on critically ill patients; one was the clinical guideline for adult patients’ sedation in ICU; two were reviews for dexmedetomidine use in children; one was a review for clinical pharmacokinetics and pharmacodynamics of dexmedetomidine. A cluster analysis of the co-citation references was performed to uncover common themes in similar articles. The co-cited references were divided into 10 visualized clusters which were labeled by the LSI algorithm with “delirium” being the most prominent cluster, followed by pharmacokinetics and pharmacodynamics, ICU sedation, critically ill patients, and nerve block (Figure 3B).

**TABLE 4:** The top 10 co-cited clinical references related to dexmedetomidine research from 2002 to 2021^a^.

Rank	Co-cited References	Title	Type	Co-cited counts	Centrality
1	Riker RR, 2009, JAMA, V301, P489, DOI 10.1001/jama.2009.56	Dexmedetomidine vs. midazolam for sedation of critically ill patients: a randomized trial	Article	89	0.22
2	Weerink MAS, 2017, CLIN PHARMACOKINET, V56, P893, DOI 10.1007/s40262-017-0507-7	Clinical Pharmacokinetics and Pharmacodynamics of Dexmedetomidine	Review	87	0.03
3	Barr J, 2013, CRIT CARE MED, V41, P263, DOI: 10.1097/ccm.0b013e3182783b72	Clinical practice guidelines for the management of pain, agitation, and delirium in adult patients in the intensive care unit	Guideline	81	0.17
4	Jakob SM, 2012, JAMA, V307, P1151, DOI: 10.1001/jama.2012.304	Dexmedetomidine vs. midazolam or propofol for sedation during prolonged mechanical ventilation: two randomized controlled trials	Article	68	0.22
5	Su X, 2016, LANCET, V388, P1893, DOI 10.1016/S0140-6736 (16)30,580–3	Dexmedetomidine for prevention of delirium in elderly patients after non-cardiac surgery: a randomised, double-blind, placebo-controlled trial	Article	59	0.03
6	Djaiani G, 2016, ANESTHESIOLOGY, V124, P362, DOI 10.1097/ALN.0000000000000951	Dexmedetomidine *versus* Propofol Sedation Reduces Delirium after Cardiac Surgery: A Randomized Controlled Trial	Article	54	0.07
7	Pratik P Pandharipande, 2007, JAMA, V298, P2644, DOI: 10.1001/jama.298.22.2644	Effect of sedation with dexmedetomidine vs. lorazepam on acute brain dysfunction in mechanically ventilated patients: the MENDS randomized controlled trial	Article	50	0.14
8	Mahmoud M, 2015, BRIT J ANAESTH, V115, P171, DOI 10.1093/bja/aev226	Dexmedetomidine: review, update, and future considerations of paediatric perioperative and periprocedural applications and limitations	Review	46	0.10
9	Reade MC, 2016, JAMA, V315, P1460, DOI 10.1001/jama.2016.2707	Effect of Dexmedetomidine Added to Standard Care on Ventilator-Free Time in Patients With Agitated Delirium: A Randomized Clinical Trial	Article	40	0.05
10	Tobias JD, 2007, PEDIATR CRIT CARE ME, V8, P115, DOI 10.1097/01.PCC.0000257,100.31779.41	Dexmedetomidine: applications in pediatric critical care and pediatric anesthesiology	Review	33	0.01

^a^ Made by Citespace, link retaining factor = 3.0, maximum links per node = 10, look back years = 5, e = 1.0, selection criteria was g-index which k = 20.

**FIGURE 4:**
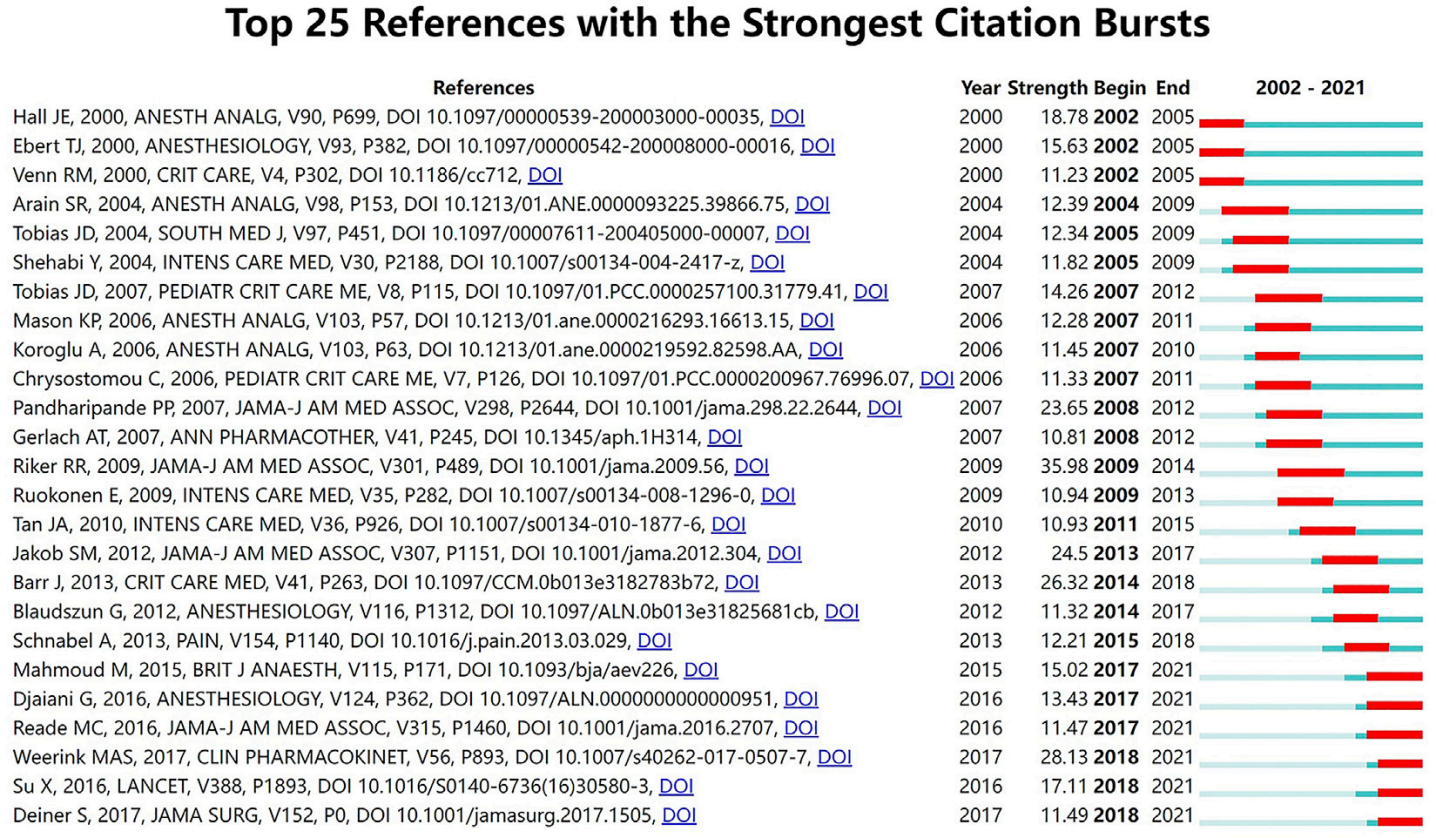
The top 25 references with the strongest citation bursts from 2002 to 2021.

Citation burstness refers to references that are often focused on closely by scholars in a specific field at an interval of time. In CiteSpace, the minimum duration of the burstness was set for 3 years and 25 references were detected with strong citation burstness from 2002 to 2021 (Figure 4). The burstness strength of the top 25 dexmedetomidine references ranged from 10.81 to 35.98, while endurance strength lasted 3–5 years. The strongest burstness (n = 35.98) among the top 10 references was caused by the paper entitled “Dexmedetomidine vs. midazolam for sedation of critically ill patients: a randomized trial” with citation burstness from 2009 to 2014.

### 3.6 Keyword detection, burst analysis, and timeline analysis

**FIGURE 5:**
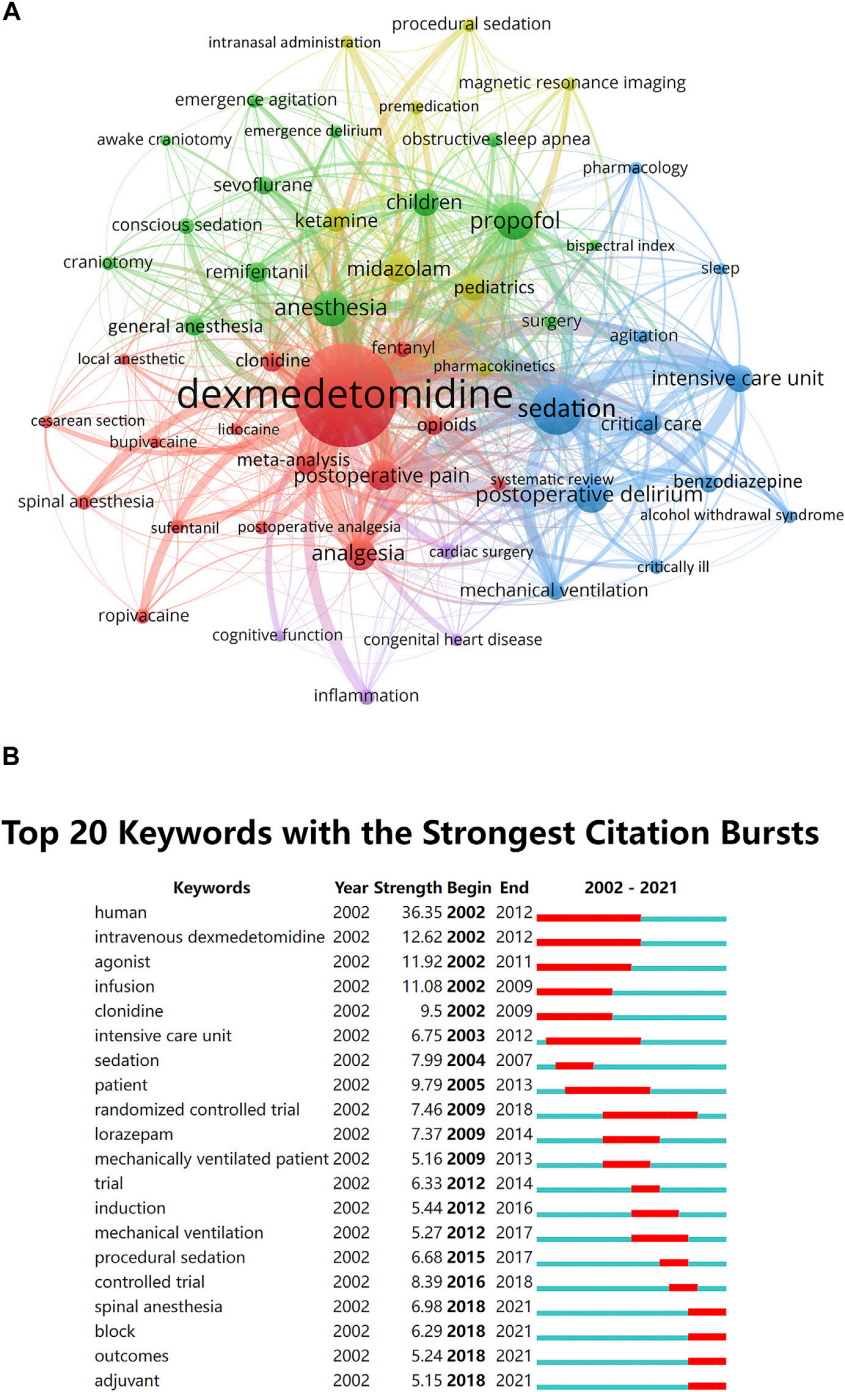
Keywords analysis. **(A)** The cluster analysis of the top 50 keywords with the highest occurrence frequencies in the dexmedetomidine area, 2002–2021 **(B)** The burst detection of keywords of dexmedetomidine area in chronological order, 2002–2021.

The top 10 keywords with the highest occurrence frequencies were dexmedetomidine, sedation, propofol, anesthesia, postoperative delirium, analgesia, postoperative pain, midazolam, children, and intensive care unit. A cluster visualization of keywords was performed with VOSviewer. With a cutoff of occurrence ≥17, the top 50 keywords were selected and five clusters emerged by co-occurrence clustering analysis (Figure 5A). The five clusters represented by different colors are as follows: 1) Premedication of dexmedetomidine in yellow: midazolam, ketamine, pediatrics, magnetic resonance imaging, and premedication, *etc.*,; 2) Intraoperative use of dexmedetomidine in green: propofol, anesthesia, children, remifentanil, general anesthesia, and sevoflurane, *etc.*,; 3) Postoperative use of dexmedetomidine in blue: sedation, postoperative delirium, intensive care unit, critical care, mechanical ventilation, and benzodiazepine, *etc.*,; 4) Analgesic effect of dexmedetomidine in red: analgesia, postoperative pain, spinal anesthesia, sufentanil, and opioids, *etc.*,; 5) Other low quantity key words in purple: inflammation, cardiac surgery, lidocaine, and cognitive function, *etc.*

The top 20 keywords with the strongest citation burstness within the last 2 decades were selected to perform a burst analysis in a 1-year slice (Figure 5B). Human, intravenous dexmedetomidine, agonist, infusion, and patient have occupied the top five positions with the highest burst strength and lasted for multiple years, suggesting intense research interests and focus. The most recent keywords with citation burstness were spinal anesthesia, block, outcomes, and adjuvant.

**FIGURE 6:**
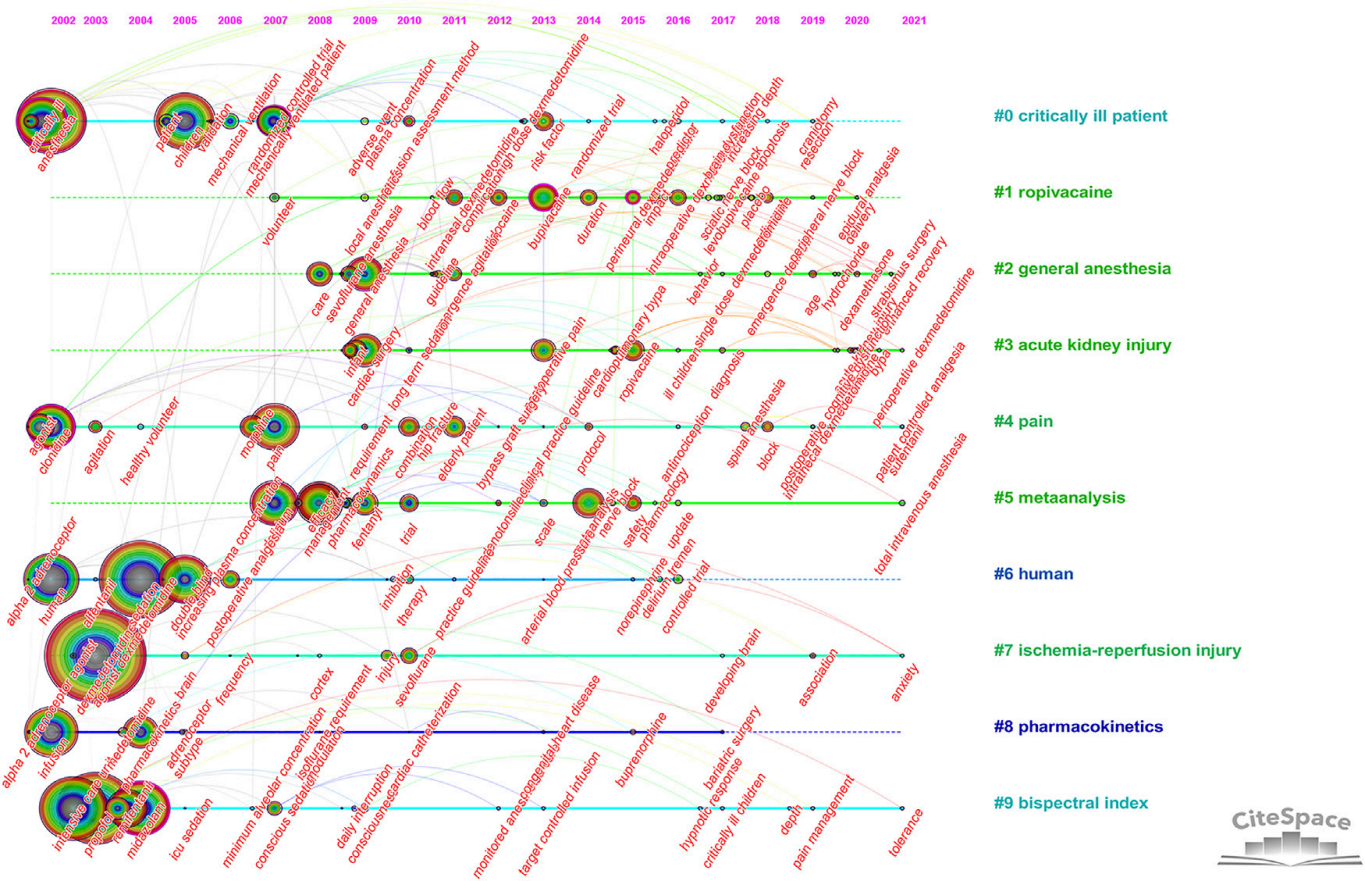
Timeline view of keywords analysis (Hot spots of one cluster were reflected by the nodes in the timeline. Clusters with more large nodes reflected the hot field of dexmedetomidine).

Timeline analysis of keywords showed the changes in hot spots of dexmedetomidine in clinical applications over time. The timeline view showed that pharmacokinetic studies on healthy humans were conducted in earlier years, pain, ischemia-reperfusion injury, and bispectral index were always hot spots since 2002, and general anesthesia and acute kidney injury were continuous research hot spots since 2008 and 2009 (Figure 6).

### 3.7 Emerging new research frontiers

Considering the time needed for the more recent publications to accumulate influence, major research advances could have been missed using TGCS alone to evaluate the importance of a publication. Therefore, we chose articles published in high impact factor (>30) journals as the secondary criterion to analyze the articles from 2017 to 2021 separately. A total of 9 records were pulled out from the *Journal of American Medical Association*, *Lancet*, *New England Journal of Medicine*, *Am J Respir Crit Care Med,* and *Intensive Care Med* (Table 5). It is worth noting that all research directions showed a high degree of consistency, that is, to explore the effect of dexmedetomidine sedation on the outcomes of critically ill patients, including delirium, morbidity, and mortality.

**TABLE 5:** Dexmedetomidine-related articles published in high impact factor (>30) journals from 2017 to 2021.

NO.	References	Title
1	Kawazoe Y, 2017, JAMA, V317, P1321, DOI: 10.1001/jama.2017.2088	Effect of Dexmedetomidine on Mortality and Ventilator-Free Days in Patients Requiring Mechanical Ventilation With Sepsis: A Randomized Clinical Trial
2	Skrobik Y, 2018, AM J RESPIR CRIT CARE MED, V197, P1147, DOI: 10.1164/rccm.201710OC-1995OC	Low-Dose Nocturnal Dexmedetomidine Prevents ICU Delirium. A Randomized, Placebo-controlled Trial
3	Collet MO, 2018, INTENSIVE CARE MED, V44, P1081, DOI: 10.1007/s00134-018-5204-y	Prevalence and risk factors related to haloperidol use for delirium in adult intensive care patients: the multinational AID-ICU inception cohort study
4	Subramaniam B, 2019, JAMA, V321, P686, DOI: 10.1001/jama.2019.0234	Effect of Intravenous acetaminophen vs. Placebo Combined With Propofol or Dexmedetomidine on Postoperative Delirium Among Older Patients Following Cardiac Surgery: The DEXACET Randomized Clinical Trial
5	Shehabi Y, 2019, N ENGL J MED, V380, P2506, DOI: 10.1056/NEJMoa1904710	Early Sedation with Dexmedetomidine in Critically Ill Patients
6	Turan A, 2020, LANCET, V396, P177, DOI: 10.1016/S0140-6736 (20)30,631–0	Dexmedetomidine for reduction of atrial fibrillation and delirium after cardiac surgery (DECADE): a randomised placebo-controlled trial
7	Hughes CG, 2021, N ENGL J MED, V384, P1424, DOI: 10.1056/NEJMoa2024922	Dexmedetomidine or Propofol for Sedation in Mechanically Ventilated Adults with Sepsis
8	Burry LD, 2021, INTENSIVE CARE MED, V47, P943, DOI: 10.1007/s00134-021-06490-3	Pharmacological and non-pharmacological interventions to prevent delirium in critically ill patients: a systematic review and network meta-analysis
9	Shehabi Y, 2021, INTENSIVE CARE MED, V47, P455, DOI: 10.1007/s00134-021-06356-8	Early sedation with dexmedetomidine in ventilated critically ill patients and heterogeneity of treatment effect in the SPICE III randomised controlled trial

Co-cited reference analysis and keyword analysis also gave hints on new research frontiers. Keyword burst analysis (Figure 5B) showed the outcome of patients and analgesic effect in nerve block and spinal anesthesia were recently widely concerned hot spots. The overall display diagram of the co-cited reference (Figure 4A) also illustrated the outcome, i.e., delirium and nerve block were the most popular topics recently. Keyword timeline (Figure 6) showed the potential organ protective property of dexmedetomidine, such as reducing acute kidney injury, had attracted researchers’ interest in recent years. All the above topics have the possibility of becoming potential research frontiers in the future.

## 4 Discussion

This bibliometric study summarized 2,299 publications of dexmedetomidine from the past 2 decades. Overall, this work summarized research status, development tendencies, and prevailing topics for dexmedetomidine as well as obtained an outline of global research on its impact.

### 4.1 Basic information

During the studied period, the number of annual publications continued to grow indicating the fast development and continuous research interest in dexmedetomidine. We predict continuous growth in the next few years. The TGCS showed a fluctuating but generally rising trend. The TGCS reached a peak in 2009 which might be associated with the SEDCOM study (Riker et al., 2009), a phase Ⅳ trial comparing dexmedetomidine and midazolam for light sedation, which found a reduction in the prevalence and duration of delirium and a significantly shorter time to extubation in the dexmedetomidine group. This trial had the highest centrality in co-cited reference analysis and led to guideline (Barr et al., 2013) from the Society of Critical Care Medicine which recommended dexmedetomidine for sedation to prevent delirium in preference to benzodiazepines. From 2018, the TGCS decreased due to the limited time for recent publications to accumulate influence, despite several notable discoveries being found.

As for the country, the United States is the absolute leader who has the most publication and the highest academic reputation. This could be due to pioneer research institutions like Harvard University, Vanderbilt University, University of California, and Duke University. It was worth mentioning that eight of the top 10 most influential authors were from institutes in the United States. The United States has a very deep foundation and influence in the research field of dexmedetomidine. However, dexmedetomidine research in China developed rapidly and contributed the most annual output in the last several years. In the Chinese Clinical Trial Registry, more than 300 dexmedetomidine research have been registered in recent 3 years. We can foresee that China have a strong presence in this area in the coming years. Regarding academic institution networks, the Chinese institutions’ network was constructed independently in the last several years and was much later than the old network of Harvard University, Vanderbilt University, and the University of Turku. This was attributed to the fact that the listing time of dexmedetomidine in China was 10 years later than that in the United States on the one hand, on the other hand, the rapid development of the quantity and quality of clinical research in China in the past decade provided a basis for the explosive growth of research in the field of dexmedetomidine.

Most publications related to dexmedetomidine were published in journals in the fields of anesthesiology and critical care, including some universally acknowledged influential journals like *Anesthesiology*, *Anesthesia and Analgesia*, *British Journal of Anaesthesia*, *Intensive Care Medicine*, and *Critical Care*, indicating dexmedetomidine was one of the hot spots in these two subjects. It is worth noting that *Pediatric Anesthesia* has the most publications. The characteristics of safety and multi-route administration of dexmedetomidine boasted increasing investigations for different uses in pediatric patients, namely, in diagnostic non-painful procedures, in painful procedures, and in surgical premedication (Mondardini et al., 2019). *JAMA* only published six articles relating to dexmedetomidine, however, all explored the impact of dexmedetomidine on the outcome of critically ill patients and were widely cited.

Keira P Mason, Richard R Riker, Joseph D Tobias, E Wesley Ely, and Yahya Shehabi who ranked as top 10 authors in both publications and co-citations have made great contributions to the research of dexmedetomidine. Eight of the 10 most co-cited authors are from the United States, which reflects the leading position of the United States in the field of dexmedetomidine research. Many authors have their own research focus. Pratik P Pandharipande is the most co-cited author with great influence on peers. He cooperated closely with Richard R Riker, E Wesley Ely, John W Devlin, and Yahya Shehabi, and launched several randomized controlled trials focusing on dexmedetomidine for sedation in ICU (Pandharipande et al., 2007; Pandharipande et al., 2010; Hughes et al., 2021). Mika Scheinin is the most productive author. He collaborating with Harry Scheinin initiated many pharmacokinetic and pharmacodynamic studies on dexmedetomidine in healthy volunteers, laying a foundation for later clinical research (Talke et al., 2005; Snapir et al., 2006; Yoo et al., 2015). Joseph D Tobias (Tobias, 2007; Venkatraman et al., 2017) and Keira P Mason (Mason et al., 2009; Mason and Lerman, 2011) bent their efforts to dexmedetomidine research in the pediatric population.

### 4.2 Research hot spots

Research hot spots are scientific topics discussed by a relatively large number of documents in a certain period. From the perspective of bibliometrics, the most frequently cited documents are usually a concentrated expression of research hot spots in this field. To a certain extent, keywords that appear more frequently also represent research hot spots. We analyzed the co-cited reference and keywords, it was found that the research hot spots in the dexmedetomidine field included pharmacokinetics and pharmacodynamics, ICU sedation and outcome, pain management and nerve block, and premedication and use in children.

#### 4.2.1 Pharmacokinetics and pharmacodynamics

The overall display diagram of the co-cited references, co-cited reference burst analysis, and keyword burst analysis showed pharmacokinetic and pharmacodynamic studies were mostly published in the early years. Hall et al. (2000), Ebert et al. (2000), and Hsu et al. (2004) investigated the responses in sedation, analgesia, cardiorespiratory effect, respiratory effect, and memory to different plasma concentrations of dexmedetomidine in healthy humans. These publications were the top three most co-cited references in the pharmacokinetic and pharmacodynamic sub-field and have laid a foundation for later research. In recent years, the studies are mainly focused on pediatric populations (Mahmoud and Mason, 2015; Morse et al., 2021), as well as on other administration routes, such as oral or nasal routes (Li et al., 2018; Chamadia et al., 2020). In 2017, Weerink et al. (2017) reviewed clinical pharmacokinetics and pharmacodynamics of dexmedetomidine in various populations *via* different administration routes, which was followed closely by the researchers from 2018 to 2021 with a citation bursts strength of 28.13.

#### 4.2.2 ICU sedation and outcome

The efficacy and safety of sedation with dexmedetomidine in critically ill patients have been a persistent hot spot. The top six most co-cited randomized clinical trials (RCT) all focused on this topic. The MENDS trial (Pandharipande et al., 2007), SEDCOM trial (Riker et al., 2009), and MIDEX and PRODEX trial (Jakob et al., 2012), all evaluated the safety and efficacy of dexmedetomidine compared with benzodiazepine or propofol and found greater benefits for the time to extubation and delirium. The results influenced the recommendations of the Society of Critical Care Medicine guidelines in 2018 (Devlin et al., 2018). The expert panel suggested that sedation strategies using dexmedetomidine may be preferred over sedation with benzodiazepines to improve clinical outcomes in mechanically ventilated adult ICU patients (Devlin et al., 2018). In 2016, another three RCTs were published and attracted wide attention with sustained citation burstnesss in the following 5 years. Su et al. (2016) found dexmedetomidine significantly decreases the occurrence of delirium in elderly patients who were admitted to the ICU after non-cardiac surgery. Djaiani et al. (2016) reported dexmedetomidine sedation reduced the incidence, delayed onset, and shortened duration of delirium in elderly patients after cardiac surgery. Reade et al. (2016) demonstrated dexmedetomidine increased ventilator-free hours among patients with agitated delirium receiving mechanical ventilation in the ICU. We collected the documents published in high IF journals from 2017–2021 (Table 5), all the articles investigated the outcomes of critically ill patients when sedated with dexmedetomidine. This topic has always been and will continue to be a hot spot.

#### 4.2.3 Pain management and nerve block

The analgesic effect of dexmedetomidine was discovered long before (Jaakola et al., 1991) and applied to clinical practice. Intravenous dexmedetomidine during surgery provided effective postoperative analgesia without increasing the incidence of side effects (Gurbet et al., 2006). A meta-analysis (Schnabel et al., 2013) with a citation burstness from 2015 to 2018 including 28 RCTs assessed the efficacy and safety of intravenous administration of dexmedetomidine compared with placebo or opioids. Dexmedetomidine led to lower postoperative pain, reduced opioid requirements, and a lower risk for opioid-related adverse events. In the last decade, the frequencies of keywords relating to spinal anesthesia, block, and adjuvant have increased, which was closely linked with an urgent need for multimodal pain therapy in the enhanced recovery after surgery (ERAS) era and the rapid development of nerve block. Perineural and intrathecal dexmedetomidine shorten the onset time, prolonged block duration, and reduced postoperative opioid consumption (Vorobeichik et al., 2017; Shen et al., 2020), leading to widespread use in various clinical scenarios. As an analgesic adjunct, dexmedetomidine also provided satisfactory effects when used for intramuscular administration (Ambesh and Dubey, 2021), intra-articular injection (Alipour et al., 2014), pediatric caudal anesthesia (Tong et al., 2014), and epidural labor analgesia (Zhang et al., 2019). Although the concept of multimodal analgesia and regional anesthetic techniques have been introduced into clinical practice, postoperative pain is still undermanaged. As a safe analgesic adjuvant, dexmedetomidine will be a hot spot in the field of pain management and nerve block in the future.

#### 4.2.4 Premedication and use in children

Despite the lack of approved pediatric labeling, contributions to the literature on clinical applications of dexmedetomidine in children have increased dramatically. As above mentioned, the journal of *Pediatric Anesthesia* published the most articles in the dexmedetomidine field. In the early years, Chrysostomou et al. (2006) and Tobias (2007)) explored the efficacy and safety of dexmedetomidine use in pediatric patients. Both articles were selected into the top 25 references with the strongest citation bursts. Similar to the research in the adult population, the studies of dexmedetomidine in children covered its pharmacokinetics and pharmacodynamics, sedative and analgesic effect, organ protective property, *etc.* However, the most distinctive feature in children is that dexmedetomidine is used for surgical premedication and diagnostic non-painful procedures since anxiolysis is an important aspect of pediatric perioperative planning and respiratory-sparing effects and bioavailability by various routes of dexmedetomidine are some of the valued features. The intranasal route is the most used extravascular route in children and a large number of relevant articles have been published (Peng et al., 2014; Mondardini et al., 2019). In 2015, Mahmoud and Mason (2015) reviewed the perioperative applications, precautions, and end-organ effects of dexmedetomidine in pediatric patients, gaining persistent attention in the following years with a citation burst from 2017 to 2021. Perioperative ‘off-label’ use of dexmedetomidine in the pediatric population is promising but still limited, and further in-depth studies are warranted.

### 4.3 Research frontiers and trends

Documents published in high IF journals from 2017–2021, keywords burst analysis and timeline analysis, and co-cited overall display diagram and burst analysis provided the clue to reveal the trend of hot subject categories and research frontiers. The use of dexmedetomidine compared to other sedatives in critically ill adults resulted in a lower risk of adverse outcomes in early RCTs (Pandharipande et al., 2007; Riker et al., 2009; Jakob et al., 2012). However, articles published in high IF journals from 2017–2021 have reported more neutral results (Kawazoe et al., 2017; Shehabi et al., 2019; Turan et al., 2020; Hughes et al., 2021). The contradictory results will promote more in-depth research in the future. The study should differentiate various procedures, populations, and settings and determine appropriate dosage, aiming at an enhanced understanding of the risk/benefit ratio to ensure patient safety. The keyword burst analysis showed spinal anesthesia, block, and adjunct were present citation bursts, illustrating the opioid-sparing analgesic effect of dexmedetomidine will still be a hot spot in the future. Clarifying the optimal doses in diverse populations and different administration routes and emphasizing safety issues are the focus of future research. Keyword timeline analysis showed the role of dexmedetomidine in reducing acute kidney injury has attracted great interest. In fact, many preclinical and clinical studies have confirmed that dexmedetomidine has a protective effect on a variety of organs, including the kidneys, nervous system, lungs, heart, liver, and small intestine (Bao and Tang, 2020). However, a majority of the current research is based on animal experiments and at the bench-top level, the mechanism is not fully elucidated. Clinical trials exploring appropriate dosage and duration are necessary to validate the potential for using dexmedetomidine to protect organs in humans.

### 4.4 Limitations

Our study has several limitations. Firstly, data were retrieved only from the WoSCC other than databases like Embase or Scopus. However, we have to note that current scientometric tools face extreme difficulties in analyzing data from multiple databases simultaneously and WoSCC is the most commonly used for scientometric analysis. Secondly, all information was extracted by scientometric tools but not manually by authors. Thus, the bias of our results may also exist. For example, the possibility of homonyms of authors would not be excluded. Lastly, the publications in 2022 were not included because of the inadequate data. The ability of mining frontiers may be weakened.

## 5 Conclusion

The annual scientific publications on dexmedetomidine increased rapidly during the last 2 decades. It is foreseeable that it will continue to increase in the coming years. In terms of clinical research in the dexmedetomidine field, pharmacokinetics and pharmacodynamics, ICU sedation and outcome, pain management and nerve block, and premedication and use in children are the mainstream hot spots. The effect of dexmedetomidine sedation on the outcomes of critically ill patients, the analgesic effect of dexmedetomidine, and its organ protective property will be the frontiers in future research.

## Data Availability

The original contributions presented in the study are included in the article/supplementary material, further inquiries can be directed to the corresponding author.

## Abbreviations

ERAS, Enhanced Recovery After Surgery; FDA, Food and Drug Administration; ICU, Intensive Care Unit; JCR, Journal Citation Reports; JAMA, Journal of the American Medical Association; SCI-EXPANDED, Science Citation Index Expanded; TGCS, Total Global Citation Score; WoSCC, Web of Science Core Collection.
